# Monthly Summary

**Published:** 1886-05

**Authors:** 


### Monthly Summary.

OXYPHOSPHATES AND TEMPERATURE.—A very troublesome obstacle to success in the use of the oxyphosphate cements will often be found in the temperature of the air, an elevated temperature so hastening those chemical changes upon which the hardening of these cements depends as to render their use almost impracticable. This difficulty is likely to occur only in the hotter seasons of the year, and can readily be overcome by placing the mixing-slab, as well as the acid and oxide bottles, in cold water until their temperature has been considerably reduced.

During severe winter weather too low a temperature also gives trouble, the acid and oxide, even when the former is in some excess, forming a powdery mass utterly unworkable, but which melts down into an almost fluid condition when brought into contact with the warmth of a tooth *in situ*. A temperature between 60° and 65° F. secures the best results in mixing oxyphosphate cements.—Litch, in *Dental Casmos.*

DISCOLORATION OF GOLD FILLINGS.—"We learn from the *Odontographic Journal* that the result of Dr. S. P. Palmer's researches into the causes of gold fillings becoming discolored seems to establish the fact that iron is the chief agent. Cabbage or other vegetables cooked in an iron pot, or water drawn from an iron tank, accounts for some cases of discoloration. Iron exhibited in combination with the mineral acids, in physician's prescriptions, may also discolor fillings. Dr. Watt once called attention to the fact that the gold-beater's vellum may communicate foreign mineral particles to gold foil.

Dr. Palmer's theory will no doubt appear more plausible to the dentist who does not believe in shouldering more than his share of responsibility."

The above is an editorial in the February number of the *Cincinnati Medical and Dental Journal.* Long ago Dr. A. M. Leslie gave his opinion, at a meeting of the Mississippi Valley Association, that a certain peculiar coloring on gold fillings was due to a trace of iron in combination with the gold. Either by appointment or private agreement it became understood that Dr. L. and the writer would give the subject special attention. We both became well convinced that Dr. L's theory was correct. When preparing gold for crystallization, in former years, we found it necessary to give special care to the removal of all traces of iron, or the coloring referred to would be found on gold fillings in all mouths containing cyanide of sulphur, sometimes called sulpho-cyanogen. We presume this is the same kind of discoloration spoken of above. It is due to iron.—*Ohio State Journal of Dental Science.*

---

DANGER FROM THE BICYCLE.—Each new development in the mechanical arts of civilization seems to bring with it its own especial disease. Even the pleasures and recreations of the student or the business man often bring, with the improvement in general health, a diseased action in some special organ which is by chance thus subject to some undue strain or irritation. The public is beginning to understand the danger of heart strain that comes to the student with the increased muscle and apparent vigor which result from the training for the college regatta.

The disease which will be induced by the use of the bicycle is not so well understood. The horseman, after his fortieth year, is apt to show symptoms of disease of the prostate gland. It is the result of the pressure of the saddle against the gland. The pressing and the jarring create an irritation, which passes into a chronic congestion, then glandular hypertrophy, with mechanical obstruction to the free escape of urine, a bladder developing a chronic cystitis from the retained and decomposing fluid, a secondary kidney affection, and death. The extra risk of development of this line of disease to the horseman is well known. Yet the saddle which he uses affords quite a broad, secure seat. If one examines the saddle or seat of the bicycle, however, he finds a narrow support of only a few inches for the whole weight of the body. A wider saddle is not possible, as it would interfere with the free use of the feet on the pedals. The horseman, upon the broad saddle, has the additional advantage that the weight of the body rests principally upon the firm tuberosities of the ischii, while with the bicyclist, owing to the narrowness of the saddle, the weight comes upon the perineum, and is transmitted directly to the prostate gland and base of the bladder. If the horseman develops a tendency beyond that of the average of men to trouble with the prostate and urinary organs, the habitual user of the bicycle must develop to a much more marked degree the same tendency, and the result of a general use of the bicycle must be an equally general tendency to hypertrophy of the prostate with its attendant ills.—*Southern California Practitioner.*

---

WHITE OF EGG IN OBSTINATE DIARRHŒA.—From *Allg. Med. Cent.-Zeit.* we learn that Celli has recently called attention to the curative properties of the albumen of hen's eggs in severe diarrhœal affections. In a discussion before a medical society at Rome, he advocated its use, and related two cases of chronic enteritis and diarrhœa which, having resisted all treatment, speedily made complete recoveries under the use of egg-albumen. The same diet is strongly recommended in the diarrhœa accompanying febrile cachexia and in that of phthisis. In two cases of diarrhœa dependent upon tertiary syphilis, it was of no avail. On post-mortem examination diffuse amyloid degeneration of the arterioles of the villi was found in these cases. The whites of eight or ten eggs are beaten up and made into an emulsion with a pint of water. This is to be taken in divided quantities during the day. More may be given if desired. The insipid taste can be improved with lemon, anise, or sugar. In case of colic, a few drops of tincture of opium may be added.—*Epitome.*

A DOCTOR'S LUCK.—The *Phila. Press* states that Dr. Thomas C. Stellwagen, a dentist of that city, and a resident of Media, Pa., has come into the possession of a handsome fortune by the death of his aunt, the widow of the late Dr. Dickey, of Atlanta, Ga. The story goes that Mrs. Dickey, assuming to be in want, addressed letters to relatives in Maryland and Pennsylvania, soliciting aid, but that Dr. Stellwagen was the only one who responded favorably. He sent her two checks for $50 each, and wrote that he hoped his small contributions would help her. Three months ago Dr. Stellwagen received a letter from his aunt saying she was very sick, and requesting him to come to Atlanta. He obeyed the summons, and when he reach Atlanta was dumbfounded to find that Mrs. Dickey was living elegantly and that she was wealthy. She told him that she wouldn't live long; that she wanted to go home with him, and it is said deeded everything to him, and made him a present of all her household furniture, silverware and jewels. It is also said that the professor left behind him in the Southern city a row of handsome dwelling-houses. Two days after reaching the Doctor's home in Media Mrs. Dickey died.

---

ARTIFICIAL IVORY.—An extensive industry has arisen in France to supply an artificial substitute for natural ivory in view of the growing insufficiency of the latter to meet the demands of art and industry. The majority of the products formerly employed were obtained by injecting whitewood with chloride of lime under strong pressure. At the Amsterdam exhibition, however, almost all the products had been prepared with the bones of sheep and waste pieces of deer and kid skins. The bones are for this purpose macerated and bleached for two weeks in chloride of lime, then heated by steam along with the skin, so as to form a fluid mass, to which are added a few hundredths of alum; the mass is then filtered, dried in the air, and allowed to harden in a bath of alum, the result being white tough plates, which are more easily worked than natural ivory.

---

REPAIRING A CRACKED PLATE.—Occasionally I have to repair a cracked plate; whenever I do, I use what is known to jewelers as earring wire—it is a brass wire gold plated—I proceed in the usual way till about to pack in the new rubber, when I make a hole sufficiently large to receive the wire each side of the crack as far away from the crack as the outer edge of the patch, then take a piece of the wire and bend one end at a right angle; get the distance between the holes and cut the wire to the proper length and bend the other end at a right angle also, making a broad staple. I put the ends in the holes, letting them go clear through the plate, unless it is quite thick then pack the rubber over and proceed as usual. If the wire projects through the plate cut or file it off.

In this way a plate may be repaired and be as thin when done as it was originally, and stronger, thus avoiding a clumsy patch. Two or more of such staples may be put in as the case demands. This method was given me by Dr. Abbott, of Dwight, Ills.—*Items of Interest.*

---

SHARPENING CORUNDUM WHEELS.—I find after using awhile, my corundum wheels seem to be glazed and do not take hold and cut as readily as they used to.

One day, when particularly bothered, I suddenly remembered that the wheels were made with shellac. Alcohol is a solvent. I placed the wheel in a horizontal position, poured a spoonful of it on, then using a stiff brush scrubbed the face of the wheel, laid it aside to dry thoroughly, and when I used it again found it nearly equal to a new one. This can be done as often as they get dull, being careful not to use till thoroughly dry.—*Items of Interest.*

---

GOLD SOLDER.—The following formulæ for gold solder is given by Dr. McKellops :

| | |
|---|---|
| Gold, . . . . . . . | 89 parts, |
| Copper, . . . . . . . . | 4 parts, |
| Silver, . . . . . . . | 7 parts. |

This solder will not show on the plate. It should be reduced in the same proportion as the gold is reduced for making it.

